# Association between self-reported race and ethnicity and myositis-specific autoantibodies in a diverse cohort of patients with inflammatory myopathy

**DOI:** 10.1007/s10067-023-06719-0

**Published:** 2023-08-04

**Authors:** Michael J. Beam, Anna Montgomery, Christine Anastasiou, Gabriela Schmajuk

**Affiliations:** 1 Department of Medicine, University of California San Francisco, San Francisco, CA USA; 2 San Francisco Veterans Affairs Health Care System, San Francisco, CA USA; 3 University of California San Francisco, San Francisco, CA USA; 4 Division of Rheumatology, Department of Medicine, University of California San Francisco, San Francisco, USA

**Keywords:** Autoantibody, Inflammatory myopathy, Myositis, Myositis-specific autoantibody, Race and ethnicity

## Abstract

Myositis-specific autoantibodies (MSAs) are highly specific biomarkers for idiopathic inflammatory myopathies (IIMs). We investigated whether self-reported race and ethnicity were associated with the presence of specific MSAs. Charts of patients with IIM seen at 3 large healthcare systems in the same US city were reviewed. Demographic data and MSA test results were abstracted. Associations between race and ethnicity and presence of MSAs were analyzed using bivariate analysis and further characterized using separate unadjusted and adjusted logistic regression models. One hundred twenty-one subjects were included (19% Asian, 10% Black or African American, 27% Latinx or Hispanic, 36% non-Hispanic White, and 7% Other). In a bivariate analysis, anti-Jo-1 and anti-MDA5 autoantibodies were associated with race and ethnicity (*p* = 0.03 and 0.02, respectively). Black or African American subjects had increased odds of a positive anti-Jo-1 result compared to non-Hispanic White subjects on unadjusted logistic regression analysis (OR 8.61, 95% CI 1.61–46.07), although after adjustment for age and gender this finding was not significant. Subjects categorized as Other had increased odds of a positive anti-MDA5 result compared to non-Hispanic White subjects on both unadjusted (OR 55.0, 95% CI 2.02–1493) and adjusted analyses (OR 44.8, 95% CI 1.55–1298). Anti-Jo-1 and anti-MDA5 autoantibodies were significantly associated with race and ethnicity on bivariate analysis. Black or African American subjects had increased odds of positive anti-Jo-1 autoantibody on unadjusted, but not adjusted, logistic regression analysis. Subjects characterized as Other had increased odds of positive anti-MDA5 autoantibody, although confidence intervals were wide.

**Table:** 

**Key Points** • *Association found between MSAs and race and ethnicity in diverse US cohort* • *Anti-Jo-1 and anti-MDA5 associated with race and ethnicity in bivariate analyses*

**Supplementary Information:**

The online version contains supplementary material available at 10.1007/s10067-023-06719-0.

## Introduction

Idiopathic inflammatory myopathies (IIMs) are a group of rare diseases that affect individuals from all backgrounds, regardless of self-reported race and/or ethnicity [1]. IIMs include dermatomyositis (DM), polymyositis (PM), anti-synthetase syndrome, immune-mediated necrotizing myopathy (IMNM), overlap syndrome, and inclusion body myositis (IBM). To varying degrees, these diseases affect skeletal muscle, smooth muscle, cardiac muscle, joints, skin, and lungs, and some IIMs have been associated with an increased risk of interstitial lung disease, dysphagia, and/or malignancy. Myositis-specific autoantibodies are highly specific biomarkers for IIMs. It is estimated that between 60 and 70% of cases of IIM are associated with one or more positive MSA [2, 3]. MSAs are not always present, but when present, they are highly specific for IIM and therefore can support diagnosis and help guide management. One MSA, anti-Jo-1, is included in the 2017 European League Against Rheumatism (EULAR) and the American College of Rheumatology (ACR) classification criteria for IIMs, and these guidelines recommend its evaluation in making the diagnosis of DM/PM [1]. In addition, because many MSAs have a distinct clinical phenotype and risk profile, they play a useful role in management and prognosis [4].

It is unknown whether the presence of MSAs is associated with race and ethnicity, and we are not aware of any prior study that has specifically addressed this in a large racially and ethnically diverse population. MSA prevalence has been shown to vary by geographic region [2, 4–6]. We hypothesized there may be an association between MSA presence and race and ethnicity. In this cross-sectional study, we reviewed the charts of all patients with IIM seen at 3 large hospitals within one US city with a racially and ethnically diverse population to assess whether self-reported race and ethnicity was associated with the presence of MSAs.

## Materials and methods

### Data source

Data were obtained by searching the electronic health record (EHR) at three large healthcare systems in the same US city. The healthcare systems included a university tertiary care medical center, a county safety net health system, and a Veterans Affairs Medical Center. The study protocol was reviewed and approved by the institutional review board (IRB) of each site (IRB # 21-33296, approved 7/23/2021). The requirement for individual subject consent was waived at all sites pursuant to applicable IRB policies.

### Study subjects

Eligible subjects were identified through the EHR if they were age 18 years or older with 2 or more clinical encounters for myositis or inflammatory myopathy as determined by ICD-9 or ICD-10 code between January 1, 2018, and December 31, 2020, for the county safety net health system and the Veterans Medical Center and between January 1, 2018, and December 31, 2019, for the tertiary care medical center.

We excluded subjects diagnosed with a form of myositis other than IIM (e.g., pyomyositis). We also excluded subjects whose diagnosis of IIM was not confirmed by a rheumatologist, dermatologist, or neurologist at their respective institution to minimize the risk of misdiagnosis. IBM was excluded because of its unique autoantibody and clinical profile.

Each subject’s chart was reviewed by 1 author (MJB) who recorded the age at diagnosis, gender, and self-reported race and ethnicity as recorded in the EHR. Subjects whose self-reported ethnicity was not Hispanic were categorized as “Asian,” “Black or African American,” or “Non-Hispanic White” based on their self-reported race in the EHR. All subjects, regardless of race, whose self-reported ethnicity was Hispanic were designated as “Latinx or Hispanic.” Subjects who identified as “Other,” “Native Hawaiian or Pacific Islander,” or who did not designate any race or ethnicity were categorized as “Other.”

We assessed whether testing was performed at any time for the following myositis-specific autoantibodies: anti-Jo-1, anti-PL-7, anti-PL-12, anti-SRP, anti-Mi-2, anti-TIF1-gamma, anti-NXP-2, anti-MDA5, anti-SAE1, and anti-HMGCR. All laboratory test results recorded in the EHR were abstracted, including results in structured fields or in scanned clinical records.

### Statistical analysis

Data were analyzed using Stata 16 (StataCorp. 2017. College Station, TX: StataCorp LLC). Descriptive statistics were used to estimate the frequency of patient demographic and clinical characteristics, autoantibody results, and autoantibody testing. Bivariate analysis using the Fisher’s exact test assessed the association between MSA presence and race and ethnicity [7]. We adjusted for false discovery rate given multiple comparisons and report in this in appendix supplementary table A. Separate unadjusted and adjusted logistic regression models were used to assess the relationship between patient characteristics and positive MSA result. Covariates in the adjusted models included age at diagnosis (<40, 40–49, 50–59, 60–69, ≥70), gender (male or female), race and ethnicity (non-Hispanic White, Black or African American, Asian, Latinx or Hispanic, or Other), and testing site (university tertiary care medical center, county safety net health system, or Veterans Affairs Medical Center). Because of sample size limitations we were unable to include additional variables such as diagnosis in the model. For MSAs that had no positive results or testing among certain race and/or ethnicity groups, we conducted a penalized maximum likelihood regression model using the Firth correction to reduce bias in maximum likelihood estimates due to separation [8]. The variance inflation factor (VIF) was used to assess multi-collinearity. VIF for each covariate included in the adjusted models ranged from 1.01 to 1.03; therefore, all covariates were kept in the model [9].

## Results

Out of the 293 potential subjects identified by the EHR search, 121 (41.3%) were found to have physician-confirmed IIM and were included in the analysis. The race and ethnicity of the total study population was 19% Asian, 10% Black or African American, 27% Latinx or Hispanic, 36% non-Hispanic White, and 7% Other (Table 1).

**Table 1:** Patient characteristics across race and ethnicity

	Total	Asian	Black or African American	Latinx or Hispanic	Non-Hispanic White	Other/unknown^+^	*p*-value*
Total	121	23	12	33	44	9	
Age at diagnosis (mean (SD))	50 (15)	52 (18)	49 (11)	45 (14)	54 (15)	54 (14)	0.67
Female	79 (65%)	18 (78%)	3 (25%)	24 (73%)	28 (64%)	6 (67%)	**0.03**
Clinical IIM diagnosis							0.98
Dermatomyositis or anti-synthetase syndrome	82 (56%)	16 (70%)	7 (58%)	24 (73%)	29 (66%)	6 (67%)	
Overlap syndrome	14 (12%)	2 (9%)	2 (17%)	4 (12%)	6 (14%)	0 (0%)	
Immune-mediated necrotizing myopathy	7 (6%)	1 (4%)	1 (8%)	1 (3%)	3 (7%)	1 (11%)	
Anti-HMGCR (statin-induced myopathy)**	11 (9%)	3 (13%)	1 (8%)	3 (9%)	3 (7%)	1 (11%)	
Polymyositis	7 (6%)	1 (4%)	1 (8%)	1 (3%)	3 (7%)	1 (11%)	

*Statistical significance was determined by Fisher’s exact test. Significant values (*p*-value < 0.05) are bolded

**Comprises subset of immune-mediated necrotizing myopathy specifically attributed to statin use

+ includes *n*=3 subjects who declined to identify their race

Among study subjects who were tested for one or more MSA, 60% tested positive for at least one MSA. On bivariate analysis, presence of anti-Jo-1 (45% in Black or African American subjects, 26% in Latinx or Hispanic subjects, 9% in non-Hispanic White subjects, and 0% in Other subjects; *p* = 0.03) and MDA5 (14% in Asian subjects, 17% in Latinx or Hispanic subjects, 10% in Black or African American subjects, 50% in Other subjects, and 0% in non-Hispanic White subjects; *p* = 0.02; Table 2) were found to be significantly associated with race and ethnicity. No significant association was found when false discovery rate was used (Supplementary Table A). Subjects who identified as Black or African American had increased odds of a positive result for anti-Jo-1 compared to non-Hispanic White subjects in an unadjusted logistic regression analysis (OR 8.61, 95% CI 1.61–46.07), although after adjustment for age and gender this finding was not significant (Table 3). In the regression models for anti-MDA5, subjects categorized as Other were found to have increased odds of a positive anti-MDA5 result compared to non-Hispanic White subjects in both the unadjusted (OR 55.0, 95% CI 2.02–1493) and adjusted models (OR 44.84, 95% CI 1.55–1298), although confidence intervals were very wide (Table 3).

**Table 2:** Proportion of patients with one or more positive myositis-specific autoantibody by type across race and ethnicity

		Total	Asian	Black or African American	Latinx or Hispanic	Non-Hispanic White	Other/unknown^+^	*p*-value*	Cramer’s *V*
Any MSA	*N* tested	103	20	11	31	34	7	0.64	0.16
	% positive	60%	65%	64%	68%	53%	43%		
Anti-Jo-1	*N* tested	103	20	11	31	34	7	**0.03**	0.33
	% positive	17%	10%	45%	26%	9%	0%		
Anti-MDA5	*N* tested	79	14	10	24	27	4	**0.02**	0.36
	% positive	11%	14%	10%	17%	0%	50%		
Anti-Mi-2	*N* tested	83	16	10	25	26	6	0.38	0.21
	% positive	13%	0%	10%	16%	19%	17%		
Anti-NXP-2	*N* tested	79	14	10	24	27	4	0.38	0.26
	% positive	7%	21%	0%	4%	7%	0%		
Anti-SRP	*N* tested	86	17	9	26	27	7	0.72	0.14
	% positive	6%	6%	0%	4%	7%	14%		
Anti-TIF1-gamma	*N* tested	75	14	10	24	24	3	0.40	0.26
	% positive	8%	21%	0%	4%	8%	0%		
Anti-HMGCR	*N* tested	18	3	3	3	9	0	0.49	0.45
	% positive	28%	33%	0%	67%	22%	0%		
Anti-SAE1	*N* tested	55	12	5	20	17	1	0.64	0.20
	% positive	2%	0%	0%	0%	6%	0%		
Anti-PL-7	*N* tested	90	17	11	26	29	7	0.58	0.22
	% positive	2%	0%	0%	0%	7%	0%		
Anti-PL-12	*N* tested	90	17	11	26	29	7	0.99	0.15
	% positive	1%	0%	0%	0%	3%	0%		

*statistical significance was determined by Fisher’s exact test. Significant values (*p*-value < 0.05) are bolded

+ includes *n*=3 subjects who declined to identify their race

**Table 3:** Association between race and ethnicity and positive anti-Jo-1 or anti-MDA5 results

Race and ethnicity	Unadjusted OR	95% CI	Adjusted OR*	95% CI
Anti-Jo-1				
Non-Hispanic White	Ref	Ref	Ref	Ref
Asian	1.15	(0.18–7.53)	1.53	(0.21–11.12)
Black or African American	**8.61**	**(1.61–46.07)**	5.33	(0.83–34.18)
Latinx or Hispanic	3.59	(0.93–15.05)	3.51	(0.75–16.45)
Anti-MDA5				
Non-Hispanic White	Ref	Ref	Ref	Ref
Asian	11.0	(0.49–246.41)	9.33	(0.42–204.90)
Black or African American	8.68	(0.61–237.02)	12.09	(0.38–388.19)
Latinx or Hispanic	12.07	(0.61–237.03)	8.11	(0.40–163.15)
Other/unknown^+^	**55.0**	**(2.02–1493)**	**44.84**	**(1.55–1298)**

*Models were adjusted for age at diagnosis (<40, 40–49–50–59, 60–69, >70) and gender (male or female). For anti-Jo-1, subjects categorized as Other were excluded from the anti-Jo-1 model due to no positive results. For anti-MDA5, both unadjusted and adjusted models were conducted using penalized maximum likelihood regression to reduce bias in maximum likelihood estimates due to separation (the non-White Hispanic reference group perfectly predicting the outcome of no anti-MDA5 positive tests). Significant values (*p* < 0.05) are bolded

+ includes *n*=3 subjects who declined to identify their race

## Discussion

In this study, we used a diverse cohort of subjects to explore the relationship between self-reported race and ethnicity and myositis-specific autoantibodies. We found that self-reported Black or African American subjects had increased odds of a positive anti-Jo-1 result compared to non-Hispanic White subjects on unadjusted logistic regression analysis. Nearly half of subjects identifying as Black or African American who were tested for anti-Jo-1 were positive, compared to only 9% of subjects identifying as non-Hispanic White. This has important clinical implications, as anti-Jo-1 is associated with a high risk of interstitial lung disease [2, 4, 10]. In fact, in our study, 94% of study subjects with a positive anti-Jo-1 autoantibody (including all 5 Black or African American subjects who were anti-Jo-1-positive) also had been diagnosed with ILD, compared to only 19% of anti-Jo-1-negative subjects.

We also found that the presence of anti-MDA5 was significantly associated with race and ethnicity on bivariate analysis, which was not surprising considering the prior studies showing higher prevalence of anti-MDA5 in East Asian populations compared to European and US populations [2, 4, 5]. On adjusted and unadjusted logistic analyses, subjects categorized as Other were found to have increased odds of a positive anti-MDA5 result compared to non-Hispanic White subjects (Table 3), although the 95% confidence interval for this finding is very wide.

Our study has several strengths. We used a diverse population of subjects that reflected the racial and ethnic composition of a large US city, allowing for a realistic assessment of the association between MSA presence and race and ethnicity.

Our study has some limitations. While our study population was larger than in many previously reported studies, our statistical power still was limited. There is some degree of diagnostic uncertainty with IIMs, although we included only subjects for whom the diagnosis of IIM was made by a specialist, which should improve the specificity of the IIM diagnosis. We also relied on the results of commercial laboratory assays to determine MSA positivity, and the accuracy of our findings is inherently dependent on the sensitivity and specificity of those assays. While we adjusted for several covariates, due to sample size limitations, we were unable to include other potentially important covariates, including diagnosis, in the model. Finally, some of our regression models revealed very wide confidence intervals reflecting the low sample sizes in some racial and ethnic groups. In addition, the adjusted *p*-values (FDR) are not significant to the <0.05 level. Nevertheless, we believe the findings reported here can be viewed as hypothesis generating and worthy of further investigation in larger cohorts.

In summary, we found a significant association between race and ethnicity for anti-Jo-1 and anti-MDA5, but not for other MSAs. Black or African American race was associated with increased odds of a positive anti-Jo-1 result on unadjusted logistic regression analysis, although this finding did not persist after adjustment for age and gender. Subjects categorized as Other had increased odds of a positive anti-MDA5 result compared to non-Hispanic White subjects on both unadjusted and adjusted analyses. Future studies should involve large, racially, and ethnically diverse study populations to better characterize the association between race and ethnicity and MSA presence. This could help to identify patient populations with increased risk of adverse clinical outcomes that are associated with certain MSAs, thereby improving care for those patients.

### Supplementary information

ESM 1
