# Supplementary material for: Association between self-reported race and ethnicity and myositis-specific autoantibodies in a diverse cohort of patients with inflammatory myopathy
Source: Clin Rheumatol. 2023 Aug 4;42(11):3043–7. doi: 10.1007/s10067-023-06719-0 (PMC10587270; doi:10.1007/s10067-023-06719-0)
Supplement: Supplementary file 1 — ESM 1 [file 10067_2023_6719_MOESM1_ESM.docx]

Association Between Self-Reported Race and Ethnicity and Myositis-Specific Autoantibodies in a Diverse Cohort of Patients with Inflammatory Myopathy

**Tables 1-3**

**Table 1.** Patient characteristics across race and ethnicity.

|  | **Total** | **Asian** | **Black or African American** | **Latinx or Hispanic** | **Non-Hispanic White** | **Other/**  **Unknown^+^** | **p-value*** |
| --- | --- | --- | --- | --- | --- | --- | --- |
| **Total** | 121 | 23 | 12 | 33 | 44 | 9 |  |
| **Age at diagnosis (mean, (SD))** | 50 (15) | 52 (18) | 49 (11) | 45 (14) | 54 (15) | 54 (14) | 0.67 |
| **Female** | 79 (65%) | 18 (78%) | 3 (25%) | 24 (73%) | 28 (64%) | 6 (67%) | **0.03** |
| **Clinical IIM diagnosis** |  |  |  |  |  |  | 0.98 |
| Dermatomyositis or anti-synthetase syndrome | 82 (56%) | 16 (70%) | 7 (58%) | 24 (73%) | 29 (66%) | 6 (67%) |  |
| Overlap syndrome | 14 (12%) | 2 (9%) | 2 (17%) | 4 (12%) | 6 (14%) | 0 (0%) |  |
| Immune Mediated Necrotizing Myopathy | 7 (6%) | 1 (4%) | 1 (8%) | 1 (3%) | 3 (7%) | 1 (11%) |  |
| Anti-HMGCR (Statin-Induced Myopathy)** | 11 (9%) | 3 (13%) | 1 (8%) | 3 (9%) | 3 (7%) | 1 (11%) |  |
| Polymyositis | 7 (6%) | 1 (4%) | 1 (8%) | 1 (3%) | 3 (7%) | 1 (11%) |  |
| * Statistical significance was determined by Fisher’s exact test. Significant values (p-value < 0.05) are bolded.  ** Comprises subset of immune mediated necrotizing myopathy specifically attributed to statin use.  + Includes n=3 subjects who declined to identify their race. | | | | | | | |

**Table 2.** Proportion of patients with one or more positive Myositis-Specific Autoantibody by type across race and ethnicity.

|  |  | **Total** | **Asian** | **Black or African American** | **Latinx or Hispanic** | **Non-Hispanic White** | **Other/**  **Unknown^+^** | **p-value*** | **Cramer’s V** |
| --- | --- | --- | --- | --- | --- | --- | --- | --- | --- |
| **Any MSA** | N tested | 103 | 20 | 11 | 31 | 34 | 7 | 0.64 | 0.16 |
|  | % positive | 60% | 65% | 64% | 68% | 53% | 43% |  |  |
| anti-Jo-1 | N tested | 103 | 20 | 11 | 31 | 34 | 7 | **0.03** | 0.33 |
|  | % positive | 17% | 10% | 45% | 26% | 9% | 0% |  |  |
| anti-MDA5 | N tested | 79 | 14 | 10 | 24 | 27 | 4 | **0.02** | 0.36 |
|  | % positive | 11% | 14% | 10% | 17% | 0% | 50% |  |  |
| anti-Mi-2 | N tested | 83 | 16 | 10 | 25 | 26 | 6 | 0.38 | 0.21 |
|  | % positive | 13% | 0% | 10% | 16% | 19% | 17% |  |  |
| anti-NXP-2 | N tested | 79 | 14 | 10 | 24 | 27 | 4 | 0.38 | 0.26 |
|  | % positive | 7% | 21% | 0% | 4% | 7% | 0% |  |  |
| anti-SRP | N tested | 86 | 17 | 9 | 26 | 27 | 7 | 0.72 | 0.14 |
|  | % positive | 6% | 6% | 0% | 4% | 7% | 14% |  |  |
| anti-TIF1-gamma | N tested | 75 | 14 | 10 | 24 | 24 | 3 | 0.40 | 0.26 |
|  | % positive | 8% | 21% | 0% | 4% | 8% | 0% |  |  |
| anti-HMGCR | N tested | 18 | 3 | 3 | 3 | 9 | 0 | 0.49 | 0.45 |
|  | % positive | 28% | 33% | 0% | 67% | 22% | 0% |  |  |
| anti-SAE1 | N tested | 55 | 12 | 5 | 20 | 17 | 1 | 0.64 | 0.20 |
|  | % positive | 2% | 0% | 0% | 0% | 6% | 0% |  |  |
| anti-PL-7 | N tested | 90 | 17 | 11 | 26 | 29 | 7 | 0.58 | 0.22 |
|  | % positive | 2% | 0% | 0% | 0% | 7% | 0% |  |  |
| anti-PL-12 | N tested | 90 | 17 | 11 | 26 | 29 | 7 | 0.99 | 0.15 |
|  | % positive | 1% | 0% | 0% | 0% | 3% | 0% |  |  |
| * Statistical significance was determined by Fishers Exact test. Significant values (p-value < 0.05) are bolded.  + Includes n=3 subjects who declined to identify their race. | | | | | | | | | |

**Table 3.** Association between race and ethnicity and positive anti-Jo-1 or anti-MDA5 results.

| **Race & Ethnicity** | **Unadjusted OR** | **95% CI** | | **Adjusted OR*** | **95% CI** |
| --- | --- | --- | --- | --- | --- |
| **Anti-Jo-1** | | | | | |
| Non-Hispanic White | ref | ref | ref | | ref |
| Asian | 1.15 | (0.18-7.53) | 1.53 | | (0.21-11.12) |
| Black or African American | **8.61** | **(1.61-46.07)** | 5.33 | | (0.83-34.18) |
| Latinx or Hispanic | 3.59 | (0.93-15.05) | 3.51 | | (0.75-16.45) |
| **Anti-MDA5** | | | | | |
| Non-Hispanic White | ref | ref | ref | | ref |
| Asian | 11.0 | (0.49-246.41) | 9.33 | | (0.42-204.90) |
| Black or African American | 8.68 | (0.61-237.02) | 12.09 | | (0.38-388.19) |
| Latinx or Hispanic | 12.07 | (0.61-237.03) | 8.11 | | (0.40-163.15) |
| Other/Unknown^+^ | **55.0** | **(2.02-1493)** | **44.84** | | **(1.55-1298)** |
| * Models were adjusted for age at diagnosis (<40, 40-49-50-59, 60-69, >70) and gender (male or female). For anti-Jo-1, subjects categorized as Other were excluded from the anti-Jo-1 model due to no positive results. For anti-MDA5, both unadjusted and adjusted models were conducted using penalized maximum likelihood regression to reduce bias in maximum likelihood estimates due to separation (the non-White Hispanic reference group perfectly predicting the outcome of no anti-MDA5 positive tests). Significant values (p < 0.05) are bolded.  + Includes n=3 subjects who declined to identify their race. | | | | | |

**Supplementary Table A.** Proportion of patients with one or more positive Myositis-Specific Autoantibody by type across race and ethnicity, with False Discovery Rate adjustment.

|  |  | **Total** | **Asian** | **Black or African American** | **Latinx or Hispanic** | **Non-Hispanic White** | **Other/Un-known^+^** | **p-value*** | **Cramer’s V** | **false discovery rate (FDR)adjusted p-value **** |
| --- | --- | --- | --- | --- | --- | --- | --- | --- | --- | --- |
| **Any MSA** | N tested | 103 | 20 | 11 | 31 | 34 | 7 | 0.64 | 0.16 | 0.78 |
|  | % positive | 60% | 65% | 64% | 68% | 53% | 43% |  |  |  |
| anti-Jo-1 | N tested | 103 | 20 | 11 | 31 | 34 | 7 | **0.03** | 0.33 | 0.17 |
|  | % positive | 17% | 10% | 45% | 26% | 9% | 0% |  |  |  |
| anti-MDA5 | N tested | 79 | 14 | 10 | 24 | 27 | 4 | **0.02** | 0.36 | 0.17 |
|  | % positive | 11% | 14% | 10% | 17% | 0% | 50% |  |  |  |
| anti-Mi-2 | N tested | 83 | 16 | 10 | 25 | 26 | 6 | 0.38 | 0.21 | 0.78 |
|  | % positive | 13% | 0% | 10% | 16% | 19% | 17% |  |  |  |
| anti-NXP-2 | N tested | 79 | 14 | 10 | 24 | 27 | 4 | 0.38 | 0.26 | 0.78 |
|  | % positive | 7% | 21% | 0% | 4% | 7% | 0% |  |  |  |
| anti-SRP | N tested | 86 | 17 | 9 | 26 | 27 | 7 | 0.72 | 0.14 | 0.79 |
|  | % positive | 6% | 6% | 0% | 4% | 7% | 14% |  |  |  |
| anti-TIF1-gamma | N tested | 75 | 14 | 10 | 24 | 24 | 3 | 0.40 | 0.26 | 0.78 |
|  | % positive | 8% | 21% | 0% | 4% | 8% | 0% |  |  |  |
| anti-HMGCR | N tested | 18 | 3 | 3 | 3 | 9 | 0 | 0.49 | 0.45 | 0.78 |
|  | % positive | 28% | 33% | 0% | 67% | 22% | 0% |  |  |  |
| anti-SAE1 | N tested | 55 | 12 | 5 | 20 | 17 | 1 | 0.64 | 0.20 | 0.78 |
|  | % positive | 2% | 0% | 0% | 0% | 6% | 0% |  |  |  |
| anti-PL-7 | N tested | 90 | 17 | 11 | 26 | 29 | 7 | 0.58 | 0.22 | 0.78 |
|  | % positive | 2% | 0% | 0% | 0% | 7% | 0% |  |  |  |
| anti-PL-12 | N tested | 90 | 17 | 11 | 26 | 29 | 7 | 0.99 | 0.15 | 0.99 |
|  | % positive | 1% | 0% | 0% | 0% | 3% | 0% |  |  |  |
| * Statistical significance was determined by Fishers Exact test. Significant values (p-value < 0.05) are bolded.  + Includes n=3 subjects who declined to identify their race. | | | | | | | | | | |
